# Supplementary figures and images for: The Neural Bases of Egocentric Spatial Representation for Extracorporeal and Corporeal Tasks: An fMRI Study
Source: Brain Sci. 2021 Jul 22;11(8):963. doi: 10.3390/brainsci11080963 (PMC8394366; doi:10.3390/brainsci11080963)

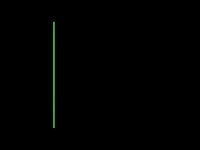

Supplement: Supplementary file 1 [file brainsci-11-00963-s001.zip › Additional file 1 SSA fMRI tasks.gif]

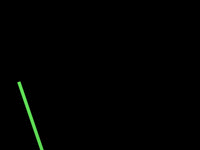

Supplement: Supplementary file 1 [file brainsci-11-00963-s001.zip › Additional file 2 SLB fMRI tasks.gif]
